# Intramedullary Screw versus Locking Plate Fixation for Traumatic Displaced Proximal Fifth Metatarsal Fractures: A Systematic Review

**DOI:** 10.3390/jcm13133952

**Published:** 2024-07-05

**Authors:** Yu-Chieh Lo, Ting-Han Tai, Yu-Min Huang, Chih-Yu Chen

**Affiliations:** 1 School of Medicine, College of Medicine, Taipei Medical University, Taipei 11031, Taiwan; b101106096@tmu.edu.tw (Y.-C.L.); b101101132@tmu.edu.tw (T.-H.T.); 2 Division of General Medicine, Department of Medical Education, Shuang Ho Hospital, Taipei Medical University, New Taipei City 23561, Taiwan; 08591@s.tmu.edu.tw; 3 Department of Orthopedics, Shuang Ho Hospital, Taipei Medical University, 291, Zhongzheng Road, Zhonghe District, New Taipei City 23561, Taiwan; 4 International Ph.D. Program in Biomedical Engineering, College of Biomedical Engineering, Taipei Medical University, Taipei 11031, Taiwan

**Keywords:** locking plate, intramedullary screw, internal fixation, proximal fifth metatarsal fractures

## Abstract

**Background/Objectives:** Intramedullary screw fixation (IMS) and locking plate fixation (LPF) are currently recommended treatments for proximal fifth metatarsal fractures (PFMF). However, treating comminuted or small displaced avulsion PFMF with IMS poses challenges due to complications. A novel alternative fixation method, the locking compression plate for distal ulna hook plate fixation (LPF), has been introduced recently for distal ulna fractures and has shown improved clinical results. This scoping review aims to assess whether LPF yields superior outcomes, such as postoperative AOFAS scores and rate of postoperative complications, compared to IMS in PFMF treatment. **Methods:** This review included randomized controlled trials (RCTs), prospective cohort studies, retrospective cohort studies, or case series involving patients with PFMF who underwent plate fixation or screw fixation. The primary outcome was the postoperative American Orthopedic Foot and Ankle Society (AOFAS) score. Studies were sourced from databases including PubMed, Embase, and Scopus, with the search conducted up to February 2024. The Systematic Review protocol was registered in the CRD PROSPERO database (CRD42024532593). **Results:** Ten studies were included, comprising 3 cohort studies, 1 case–control study, and 6 case series, with a total of 309 patients (158 with LPF and 142 with IMS). The postoperative AOFAS scores showed no significant difference between LPF and IMS in treating PFMF. However, LPF demonstrated efficient surgical procedures and enhanced functional outcomes. Complications were minimal in both groups, with no significant difference in the rate of postoperative complications. **Conclusions:** Although there was no significant difference in AOFAS scores between LPF and IMS, LPF demonstrated efficient surgical procedures and enhanced functional outcomes, making it a reasonable alternative method for PFMF. Effective shared decision-making (SDM) with patients becomes paramount in choosing the optimal surgical approach. In the surgical landscape, thoughtful deliberation, patient engagement, and adherence to biomechanical principles are crucial for achieving successful outcomes in the treatment of PFMF.

## 1. Introduction

Fifth metatarsal fractures, occurring more frequently than other metatarsal fractures, particularly at the base, are classified into three anatomical zones, with the base defined as the proximal 1.5 cm of the shaft distal to its articular surface, and are most commonly represented by proximal fifth metatarsal fractures (PFMF) [1]. Fifth metatarsal fractures are categorized into metatarsal base fractures (zone 1, zone 2, zone 3), shaft fractures, dancer’s fractures, and stress fractures. Lawrence and Botte [2] introduced the proximal fifth metatarsal fracture classification system, where Zone 1 involves avulsion of the cancellous tuberosity by the lateral band of the plantar fascia, Zone 2 extends obliquely proximal to the fourth and fifth metatarsal articulations, and Zone 3, often stress fractures, occurs just distal to the fourth and fifth metatarsal articulations. Numerous fifth metatarsal fractures, such as Jones fractures involving the metaphyseal-diaphyseal junction extending into the articulation between the fourth and fifth metatarsals, manifest with lower force than anticipated, indicating an underlying stress fracture and presenting a relatively elevated likelihood of delayed union or non-union when managed non-operatively [3]. The benefits of surgical fixation compared to non-operative treatment have been extensively documented [4].

In proximal fifth metatarsal fractures (PFMF), intramedullary screw (IMS) fixation is widely utilized. Previous studies recommend using larger screws, and athletes with acute Jones fractures can safely resume play after intramedullary screw fixation. The return-to-play (RTP) outcomes after surgically managing Jones fractures in athletes were excellent, regardless of the implant or sport. Intramedullary screw fixation outperformed nonoperative management, showing a higher RTP rate, shorter time to RTP, increased union rate, and improved functional outcomes [5,6,7,8]. However, treating comminuted or small displaced avulsion proximal fifth metatarsal fractures (PFMF) with IMS posed challenges, and outcomes were unsatisfactory due to delayed weight-bearing [9].

The novel alternative fixation method, the locking compression plate distal ulna hook plate fixation (LPF), was introduced recently for treating distal ulna fractures [10]. Moreover, studies involving the use of a locking compression plate distal ulna hook plate fixation in case series have shown improved clinical results, confirming its suitability and reliability for achieving anatomical alignment and secure stabilization of zone 1 or 2 fractures in the base and tuberosity of the fifth metatarsal [10,11]. This emphasizes the positive outcomes and safety linked to this approach.

To our knowledge, while some clinical studies have compared outcomes for various techniques in proximal fifth metatarsal fractures (PFMFs) [12,13,14,15,16], there is no definitive evidence comparing clinical, functional, and patient satisfaction outcomes between intramedullary screw (IMS) and locking compression plate (LCP) distal ulna hook plate (LPF) treatments for PFMFs. Therefore, the aim of this systematic review is to assess whether LCP distal ulna hook plate fixation yields superior outcomes compared to IMS in all zones of PFMF treatment. The population (P) included patients diagnosed with proximal fifth metatarsal fractures who required surgical intervention. The intervention (I) was the use of locking compression plate distal ulna hook plate fixation (LPF), a novel method introduced for treating distal ulna fractures, now adapted for PFMF. The comparator (C) was intramedullary screw fixation (IMS), a commonly recommended treatment but often challenging in cases of comminuted or small displaced avulsion fractures due to complications. The outcomes (O) assessed were primarily the postoperative American Orthopedic Foot and Ankle Society (AOFAS) scores, indicating pain and functional status, and secondarily, the time to union, rate of postoperative complications, and overall patient satisfaction.

## 2. Materials and Methods

### 2.1. Inclusion Criteria

A study was eligible for inclusion in this review if it satisfied the following criteria: (1) being a randomized controlled trial (RCT), prospective cohort study, retrospective cohort study, or case series; (2) involving patients with proximal fifth metatarsal fractures who underwent plate fixation or screw fixation as the exposure of interest; (3) examining the clinical outcome of fixation following proximal fifth metatarsal fractures; and (4) clearly delineating criteria for the inclusion or exclusion of patients, surgical procedures, and the definitions and assessments of outcome measures. Research that focused on examining biomechanical results, was not available in English, or featured patients who were not diagnosed with proximal fifth metatarsal fractures was excluded.

### 2.2. Search Strategy and Study Selection

Studies relevant to the topic, published before February 2024, were sourced from databases including PubMed, Embase, and Scopus. The following Medical Subject Headings (MeSH) terms were used: screw fixation OR plate fixation AND proximal fifth metatarsal fracture OR jones fracture. All abstracts, studies, and citations retrieved were reviewed. Furthermore, we scrutinized the reference lists of relevant papers to identify studies meeting our criteria. The Systematic Review protocol was registered in the CRD PROS-PERO database (CRD42024532593).

### 2.3. Data Extraction

Two separate reviewers extracted both baseline and outcome data. Furthermore, details such as the first author’s name, publication year, country, sample size, participants’ ages, classifications of proximal fifth metatarsal fractures (PFMF), surgical interventions, and perioperative outcomes were also gathered. Any discrepancies were resolved through mutual discussion and double-checking of the original articles. The characteristics of each study are summarized in Table 1.

### 2.4. Methodological Quality Appraisal

The quality assessment of the incorporated studies involved scrutinizing aspects of their research design that could potentially introduce bias. This examination encompassed measures susceptible to measurement bias, inadequate accounting for confounding factors, and loss of follow-up in observational studies. Pre-intervention, at-intervention, post-intervention, and overall biases were assessed utilizing the Risk of Bias in Nonrandomized Studies of Interventions tool for cohort and case–control studies [26]. For case series research, the Joanna Briggs Institute Checklist was employed [27]. Following independent ratings by investigators, any disagreements were resolved through discussion.

### 2.5. Outcomes

The main outcomes of interest were the postoperative AOFAS score, ranging from 0 to 100 points, where higher scores indicate less pain and better function. Other outcomes assessed included the Visual Analog Scale (VAS) for pain, time to return to sport, duration until return to competitive play, as well as SF-12 physical and mental component scores as well as complications.

## 3. Results

**Figure 1:**
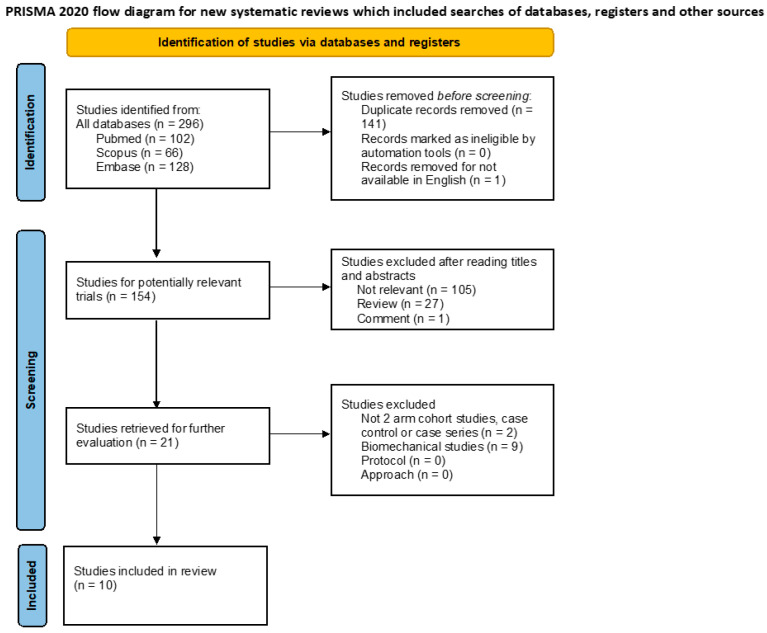
Flowchart of the selection of the included studies.

The details of the study identification and selection process are outlined in Figure 1 [28]. Initially, 296 studies were identified. After removing duplicate records and those not available in English, 154 records underwent screening based on their title and abstract. Among these, 105 studies were excluded as they were not relevant to the topic, 27 were reviews, and one was a comment. Following this, the remaining 21 records underwent a full-text review. Of these, two studies were excluded for not being two-arm cohort studies and nine were biomechanical studies. Finally, ten studies [10,17,18,19,20,21,22,23,24,25] were included in our systematic review.

### 3.1. Study Characteristics

Among the 10 included studies, 3 were cohort studies, 1 was a case–control study, and the remaining 6 were case series (Table 1). A combined total of 309 patients from these studies were chosen, comprising 158 patients who underwent treatment with LPF and 142 patients who received IMS for traumatic PFMF. The mean patient age varied from 17 to 69 years in these studies. Follow-up time ranges from 11 to 13 months. All studies provided either the score of postoperative AOFAS or time to return to competitive play. Additional details are provided in Table 2.

### 3.2. IMS

Studies regarding screw fixation show significant improvements in AOFAS scores from preoperative to postoperative (*p* < 0.001) [17,24]. Also, there are significant differences in AOFAS scores between conservative and surgical treatment groups, favoring surgical treatment with the Herbert screw (79 vs. 100, *p* < 0.001) [20].

Regarding other results and complications, Chopra (2023) [17] reported significant improvements in VAS pain (*p* < 0.0001) and AOFAS scores (*p* < 0.0001) from preoperative to postoperative. There were no significant differences in postoperative VAS pain (*p* = 0.9702), postoperative AOFAS (*p* = 0.6035), overall change in VAS pain (*p* = 1.0), or overall change in AOFAS (*p* = 0.6655) between the two groups. Additionally, only three complications (3.5%) were noted among patients who received JSI fixation, including two patients requiring the removal of symptomatic hardware. Similarly, Demel (2023) [20] found no complications in the surgical group with the Herbert screw, while one complication (deep vein thrombosis) occurred in the conservative group. Nagi (2021) [24] observed improved Short Form 12 Physical and Mental Survey scores postoperatively and satisfactory union rates in patients who underwent headless compression screw fixation. These common points highlight significant improvements in functional scores, the effectiveness of surgical interventions, and positive postoperative outcomes with minimal complications in all three studies.

### 3.3. LPF

Among studies using a locking compression distal ulna hook plate in fracture of the fifth metatarsal base, a case–control study conducted in Korea [18] found that the mean AOFAS score at 12 months after surgery showed no significant differences (*p* = 0.75) between the two groups, with scores of 97.7 ± 3.4 for the screw group and 98.2 ± 3.2 for the LPF group. Another retrospective cohort [19] reported that AOFAS scores were significantly higher in patients with LCP distal ulna hook plate fixation than the IMS cohort (*p* < 0.0001). However, some other studies all reported significant improvements in mean AOFAS scores 1 year postoperatively [10,21,22].

Regarding return to sport, Bernstein (2018) [23] noted that patients undergoing plantar plating returned at 12 weeks, while Young (2020) [25] found that most patients returned to competitive play at an average of 22.2 ± 4.5 weeks (range: 12–40 weeks). Complications included delayed union (developed in 2 patients in the LPF group and 3 in the IMS group [19]), hardware irritation [21], impaired wound healing, recurrent non-union, and persistent intermittent pain [22]. No complications, including infection, wound problems, delayed union, nonunion, incisional complications, refractures, or hardware loosening, were seen in the remaining three studies [10,18,23].

### 3.4. Quality Assessment

The Risk of Bias in Nonrandomized Studies of Interventions (ROBINS-I) tool was used for cohort and case–control studies, and all four studies [17,18,19,20] were assessed as having moderate risk of bias (Supplementary Table S1). Among the six case-series [10,21,22,23,24,25] studies, the Joanna Briggs Institute Checklist was employed, and all studies were termed low-bias quality (Supplementary Table S2).

## 4. Discussion

In our study, the comprehensive synthetic analysis revealed no significant difference in the AOFAS score following the treatment of proximal fifth metatarsal fractures (PFMF) between LPF and IMS. Conversely, a limited number of postoperative complications, such as infection, wound issues, delayed union, nonunion, refractures, or hardware loosening, were observed. Importantly, there was no significant difference in the occurrence of complications after surgery between the two cohorts [18,19]. Only three complications (3.5%) occurred, with two patients requiring the removal of plates at 4 and 6 months postoperatively for symptomatic hardware. Another patient had the JSI removed due to symptomatic hardware at 14 months postoperatively [17]. Furthermore, LCP distal ulna hook plate fixation demonstrated efficient surgical procedures and enhanced functional outcomes. The use of a locking compression distal ulna hook plate for PFMF emerges as a reasonable and alternative method, offering a shorter time to union with minimal complications.

First described by Kavanaugh et al. in 1978, intramedullary screw fixation is still indicated for proximal fifth metatarsal fractures. The simple percutaneous technique allows stable fracture fixation and, thus, early postoperative weight-bearing and a rapid return to competitive sport [29,30]. The JSI is a specialized tapered implant crafted to rectify deficiencies by closely conforming to the natural anatomy of the fifth metatarsal. Operative intervention has led to significant enhancements, accompanied by a minimal incidence of complications associated with the JSI [17]. Intramedullary screw fixation is presently the favored surgical approach for proximal metatarsal fractures, with generally positive outcomes reported in several studies [31,32]. Intramedullary Herbert screw fixation is recommended as the preferred method for treating fifth metatarsal Jones fractures due to its superior healing rates, treatment success, and minimal complications [20]. Furthermore, while low-profile “hook” plates present a viable option for stabilizing fifth metatarsal Jones fractures, IMS may offer superior resistance to bending forces and the initiation of fracture site rotation [15].

In the remaining literature review, the following findings are similar to our study results: The benefits of IMS include an average time to bone union of 8.5 weeks, a return to sport at 8.7 weeks, and an average reported midfoot AOFAS functional score of 98.4 [33]. Attia (2021) [8] provided an updated review of the return-to-play (RTP) rate and time to RTP after Jones fractures in athletes and showed IMS was superior to nonoperative management, as it led to a higher rate of RTP, a shorter time to RTP, a higher rate of union, a shorter time to union, and improved functional outcomes. However, adding to the complexity of utilizing screw fixation for proximal metatarsal fractures, cannulated screws have been linked to breakage issues owing to their insufficient strength to endure the significant forces exerted on the lateral column of the foot [34]. Additionally, despite reported surgical outcomes, complications in the form of diaphyseal stress fracture, thermal necrosis of the skin, irritation of soft tissue, non-union, and refracture were reported, which may necessitate re-operation [33,35]. The collective findings regarding IMS in proximal fifth metatarsal fractures (PFMF) indicate a 10% overall rate of metal removal, with the potential for almost a 50% rate of secondary surgery to address painful screws [36].

Initially, in 1948, Zuelzer described the use of a “hook plate” for the internal fixation of small bone fragments, which was published in 1951 [37]. In 2003, Carpenter first described this method of fixation for proximal fifth metatarsal fractures [38]. Recently, the locking compression plate (LCP) distal ulna hook plate was introduced for the treatment of distal ulna fractures. The fifth metatarsal base and its tuberosity have an anatomical architecture similar to that of the distal ulna metaphysis and its styloid process. Advantages of using this plate include angular stable fixation of the fragments, regardless of bone quality, and a lower risk of primary and secondary loss of reduction [10].

LCP distal ulna hook plate fixation offers swift surgery and improved function. It presents several advantages, including secure grip on the fifth metatarsal tuberosity, fracture stabilization, prevention of joint surface collapse, good histocompatibility, alignment with natural curvature, and reduced soft tissue irritability [15]. Given the drawbacks of intramedullary screws, tension-sided plantar plating offers advantages, enhancing fixation in the proximal metaphyseal fragment with both locking and non-locking screws. In biomechanical analysis, plantar plating outperformed intramedullary screw fixation in resisting tensile forces, as demonstrated in cadaver models subjected to cantilever bending. Additionally, contoured plates tailored to the fifth metatarsal flare address concerns about optimal diameter, maximizing screw purchase, and minimizing the risk of cortical perforation [17]. Furthermore, the authors have illustrated, through initial short-term clinical and radiographical results, that plantar plating of the proximal fifth metatarsal proves to be a safe and effective approach suitable for both revision and primary scenarios for challenging fractures. This technique facilitates direct visualization and reduction in the fracture, provides exposure for bone grafting or biologic augmentation, and allows for the application of the plate with resistance to the tensile forces on the plantar surface of the bone [39]. Additionally, with a follow-up of at least one year, the outlined plantar plating technique could serve as an alternative approach for surgically treating fifth metatarsal stress fractures, addressing concerns related to nonunion [25]. As importantly, a study on the topographical anatomy of the peroneus brevis tendon footprint used in PFMF found that hook plate implantation is safe when placed strictly laterally at the proximal aspect of the fifth metatarsal [40].

In the realm of biomechanical studies, there is substantial evidence supporting the use of plate fixation for the management of fifth metatarsal base fractures. This method has been shown to be effective in both acute and revision surgeries, particularly for comminuted fractures [41]. Three studies have specifically investigated the outcomes of surgical treatment for zones I and II fifth metatarsal base fractures using a mini-hook plate, highlighting the favorable biomechanical outcomes associated with this approach [10,21,42]. Additionally, a study comparing IMS and low-profile plate fixation for Jones fractures has demonstrated the biomechanical advantages of plate fixation in the context of fifth metatarsal fractures [15]. Utilizing plantar lateral plating proved to be an effective method for surgically addressing fifth metatarsal stress fractures. However, caution is advised as returning to activity too soon may contribute to refracture, as evidenced by a reported refracture rate of 10.5% in their retrospective series focusing on athletic stress fractures [25].

In a direct comparison of IMS and LPF, a case–control study conducted in Korea found that the mean AOFAS score at 12 months after surgery showed no significant differences (*p* = 0.75) between the two groups, with scores of 97.7 ± 3.4 for the screw group and 98.2 ± 3.2 for the LPF group [18]. However, a retrospective cohort of 43 PFMF patients [19] reported that the AOFAS scores at 9 months (82.06 and 78.64, *p* < 0.0001) and 12 months (93.56 and 87.8, *p* < 0.0001) after surgery were significantly higher in patients with LCP distal ulna hook plate fixation than in the IMS cohort. This significant difference may be attributed to all cases being performed by a single senior surgeon using the same surgical approach. However, the sample size was small, and the radiographic evaluation was incomplete.

The limitations of this study, including the study design being a scoping review, were that meta-analysis was not performed due to inadequate data and discordant research designs among the included studies, so the credibility might be finite. Additionally, the study’s postoperative outcomes were limited to twelve months. However, our study possesses strengths. Firstly, complications were minimal in the studies we included. Secondly, the methodological quality assessment of the included studies indicated a low to moderate level of bias. Nevertheless, LPF seems to be an effective and safe treatment modality compared to IMS, according to its efficient surgical procedures and enhanced clinical outcomes.

## 5. Conclusions

In conclusion, our study found no significant difference in AOFAS scores between LPF and IMS in treating proximal fifth metatarsal fractures (PFMF). Despite this, LPF exhibits distinct advantages and holds promising prospects. Effective shared decision-making (SDM) with patients becomes paramount in choosing the optimal surgical approach. In the surgical landscape, thoughtful deliberation, patient engagement, and adherence to biomechanical principles are crucial for achieving successful outcomes in the treatment of proximal fifth metatarsal fractures.

## Figures and Tables

**Table 1:** Characteristics of the included studies.

Study (Year)	Country	Study Design	Intervention and Number of Patients (Male %)	Age, Years, Mean ± SD (Range)	Zones of Injury (%)	Clinical Outcome Measurements	Conclusions
Chopra, 2023 [17]	USA	Retrospective cohort	* JSI: 12 (85.9) Plate and intramedullary screw: 73 (49.3)	49.0 ± 16.3	I, 4(4.7); II, 54(63.5); III, 27(31.8)	The total cohort showed significant improvements in VAS pain (*p* < 0.0001) and AOFAS scores (*p* < 0.0001) from preoperative to postoperative. There were no significant differences in postoperative VAS pain (*p* = 0.9702), postoperative AOFAS (*p* = 0.6035), overall change in VAS pain (*p* = 1.0), and overall change in AOFAS (*p* = 0.6655) between the two groups.	JSI was an effective and safe technique at short- to mid-term follow-up that compared favorably to current existing treatment modalities for proximal metatarsal fracture, including plating options and intramedullary screw fixation.
Kim, 2017 [18]	Korea	Retrospective case–control	Group A-headless cannulated screw for bicortical internal fixation: 15 (40) Group B-locking compression distal ulna hook plate: 15 (33)	Group A: 47 Group B: 50	I	Clinical Outcomes: The mean AOFAS score at 12 months after surgery showed no significant differences (*p* = 0.75) between the 2 groups in group A (97.7 ± 3.4) and in group B (98.2 ± 3.2). No complications, including infection, wound problem, delayed union, or nonunion, were seen in the 2 groups. Radiologic Outcomes: The displacement of diastasis decreased significantly in both groups (*p* < 0.001). Postoperative differences between groups were not statistically significant (*p* = 0.10). The reduction distance was significantly shorter in Group A (*p* = 0.04). The interval to union was significantly shorter in Group B (*p* = 0.01). Gender and age were not influential, but a greater reduction distance significantly decreased the interval to union (*p* = 0.04).	Locking compression distal ulna hook plate for fixation of zone 1 fracture of the fifth metatarsal base is a reasonable and alternative method that can provide a shorter time to union without complications.
Xie, 2017 [19]	China	Retrospective cohort	IMS: 25 (60) PF: 18 (27.8)	IMS: 34.36 ± 1.977 PF: 39.89 ± 2.739	I	Surgery time, partial weight-bearing, full weight-bearing, and bony union times were significantly shorter in the LCP cohort (*p* < 0.001, *p* < 0.001, *p* < 0.001, *p* = 0.0053). Time to return to daily life, pain scores before and after surgery, and AOFAS scores before and at 3 and 6 months were not significantly different between cohorts. AOFAS scores were significantly higher at 9 and 12 months in the LCP cohort (*p* < 0.0001). No significant difference in complications was observed.	LCP distal ulna hook plate fixation as an alternative fixation method was better therapy for the displaced avulsion FMBFs compared to IMS.
Demel, 2023 [20]	Czech	Prospective cohort	Conservative-Walker orthosis: 9 (89) Surgical-Herbert screw: 15 (80)	Conservative: 32 Surgical: 27	II	The differences in healing between the groups were significant for both X-ray (*p* = 0.022) and AOFAS scores (*p* < 0.001) at six weeks. No complications were recorded in the surgical group, whereas one deep vein thrombosis occurred in the conservative group.	Herbert screw is superior to conservative treatment for Jones fractures, offering a better healing rate after six weeks, 100% treatment success, immediate limb loading, and no complications.
Choi, 2013 [21]	Korea	Case series	mini-hook plate (Locking Compression Plate): 17	46 ± 16	I, 6; II, 17	AOFAS midfoot scale score improved from a mean of 48 preoperatively to 91 at 1 year postoperatively. The mean time to bony union was 54 days, with all patients returning to prior activities within 74 days. The only complication was hardware irritation in 1 patient.	The mini-hook plate fixation method is recommended as an effective alternative to rigid stabilization for these fractures.
Ismat, 2019 [22]	Germary	Case series	Ulnar hook plate: 21	38.7	I: 11; II: 8; III: 2	Average time to return to ADLs for all patients (*n* = 21) was 10.3 weeks (range 4.5–37 weeks), with the primarily surgically treated group (n = 18) returning in 8.1 weeks (range 4.5–21 weeks). Preoperatively, the average AOFAS midfoot score for all patients significantly improved to 95.2 postoperatively (*p* < 0.01), while in the primarily surgically treated group, it improved to 97.8 postoperatively (*p* < 0.01). Complications included impaired wound healing and recurrent non-union (1) and persistent intermittent pain (1).	The use of the ulna hook plate seems to be suitable and adequate as an osteosynthesis method to primarily treat proximal fifth metatarsal fractures requiring surgical intervention with satisfactory postoperative outcomes.
Lee, 2014 [10]	Korea	Case series	LCP distal ulna hook plates: 19	43 (18–69)	I: 12; II: 7	The mean AOFAS midfoot score improved from 26 to 94 points postoperatively (*p* < 0.01), with excellent outcomes in 84% of patients. Mild degenerative changes occurred in 16% of patients, with only 10% showing symptoms; no fixation loss or implant failure was observed. Patients resumed sports and daily activities within approximately 11.2 weeks’ post-surgery, starting partial weight bearing at 3.6 weeks and full weight bearing at 6.6 weeks.	The LCP distal ulna hook plate effectively stabilizes fractures at the fifth metatarsal base and tuberosity, offering a reliable and safe method for achieving anatomical reduction and stable fixation, with promising outcomes.
Bernstein, 2018 [23]	USA	Case series	Plantar plating: 8 (100)	21.9 ± 1.9	-	In a study of 8 male athletes (mean age: 21.9 ± 1.9 years; mean follow-up: 3.2 ± 0.4 years), temporary sural nerve neuropraxia resolved within 6 weeks for 2 patients. No complications occurred, with all athletes returning to sport at their previous level after asymptomatic radiographic union at 6.5 ± 1.1 weeks and full release at 12.3 ± 1.9 weeks.	With a minimum 2-year follow-up, plantar plating of proximal fifth metatarsal fractures was an effective and safe technique that was used in both primary and revision settings.
Nagi, 2021 [24]	UK	Case series	Headless compression screw fixation: 24 (66)	37.2 (19–56)	II	All patients achieved radiological union at a mean of 7.2 weeks. At 12-month follow-up, the mean AOFAS midfoot score was 95.6. Additionally, Short Form 12 Physical and Mental Survey scores improved from preoperative values of 22.71 and 29.31 points to 57.88 and 59.54 points, respectively.	The headless compression screw achieved a satisfactory union rate for delayed union Lawrence zone II fractures of the base of the fifth metatarsal with satisfactory functional results.
Young, 2020 [25]	Korea	Case series	Plantar plating-refracture group: 4 Plantar plating-union group: 34	Refracture group: 17.8 ± 1.7 Union group: 19.9 ± 4.0	-	The mean time to radiological union was 9.3 weeks (range: 8–16). Although no nonunions or delayed unions were observed during follow-up, 4 refractures occurred (10.5%). Most patients (all but 1) resumed previous sporting activities at 22.2 ± 4.5 weeks (range: 12–40).	With a minimum 1-year follow-up, the described plantar plating technique could be an alternative method for the operative treatment of fifth metatarsal stress fractures without nonunion problems.

VAS: Visual Analog Scale; AOFAS: American Orthopedic Foot and Ankle Society; JSI: Jones surgical intervention; LCP: locking compression plate; PF: plate fixation; IMS: intramedullary screw; FMBFs: fifth metatarsal base fractures; ADL: activities of daily living. * A Jones-specific implant is a specific curved intramedullary screw.

**Table 2:** Treatment outcomes of screw and locking plate groups.

Study, Year	Intervention (Number)	Timing of Postoperative AOFAS Score	Postoperative AOFAS, Mean ± SD (Range)	Others
Chopra, 2023 [17]	JSI (12)	1 year	87.7 ± 6.9	VAS, mean ± SD: 1.4 ± 1.6
	Others (73)		85.9 ± 11.6	VAS, mean ± SD: 1.4 ± 1.7
Kim, 2017 [18]	Screw (15)	1 year	97.7 ± 3.4	
	Plate (15)		98.2 ± 3.2	
Xie, 2017 [19]	IMS (25)	1 year	87.8 ± 0.17	
	PF (18)		93.56 ± 0.25	
Demel, 2023 [20]	Conservative (9)	1 year	79 (56–97)	
	Surgical (15)		100 (79–100)	
Choi, 2013 [21]	mini-hook plate (Locking Compression Plate)	1 year	91 ± 7 (85–100)	Return to ADL, d: I, 73 ± 14 (60–98); II, 75 ± 7 (65–84); all, 74 ± 10 (63–98)
Ismat, 2019 [22]	Ulnar hook plate	1 year	97.8 (80–100)	
Lee, 2014 [10]	LCP distal ulna hook plates	-	94 (range 72 to 100)	
Bernstein, 2018 [23]	Plantar Plating	-		Return to Sport: 12 weeks
Nagi, 2021 [24]	Headless compression screw fixation	1 year	95.6 (82.4–100)	SF-12 Physical score: 22.71 points preop (range, 7–36) to 57.88 (range, 52–64) postop. SF-12 Mental score: 29.31 points preop (range, 11–34) to 59.54 (range, 51–63) postop.
Young, 2020 [25]	plantar plating	-		Return to competitive play: 17.5 ± 3.8 weeks
	plantar plating, union group	-		Return to competitive play: 22.9 ± 4.3 weeks

JSI: Jones-specific implant; IMS: intramedullary screw; PF: plate fixation AOFAS: American Orthopedic Foot and Ankle Society score; SD: standard deviation; VAS: Visual Analog Scale pain score; d: days; wks: weeks.

## Data Availability

All data are publicly available.

## Supplementary Materials

The following supporting information can be downloaded at: https://www.mdpi.com/article/10.3390/jcm13133952/s1, Supplementary Table S1: Quality assessment of cohort and case-control studies using ROBINS-I Scale. Supplementary Table S2: Quality assessment of case series using JBI.
