# Supplementary material for: Intramedullary Screw versus Locking Plate Fixation for Traumatic Displaced Proximal Fifth Metatarsal Fractures: A Systematic Review
Source: J Clin Med. 2024 Jul 5;13(13):3952. doi: 10.3390/jcm13133952 (PMC11242427; doi:10.3390/jcm13133952)
Supplement: Supplementary file 1 [file jcm-13-03952-s001.zip › Supplementary Table S1.pdf]

Supplementary Table S1. Quality assessment of cohort and case-control studies using ROBINS-I Scale

Author, year	Preintervention		At-intervention	Postintervention				Overall
	Confounding	Selection bias	Classification of intervention	Intended interventions	Missing data	Measurement of outcomes	Reported results	Risk of bias
Chopra, 2023	Moderate	Low	Low	Low	Low	Low	Low	Moderate
Kim, 2017	Moderate	Moderate	Low	Low	Low	Low	Low	Moderate
Xie, 2017	Moderate	Low	Low	Low	Low	Low	Low	Moderate
Demel, 2023	Moderate	Low	Low	Low	Low	Low	Moderate	Moderate
