# Supplementary material for: Intramedullary Screw versus Locking Plate Fixation for Traumatic Displaced Proximal Fifth Metatarsal Fractures: A Systematic Review
Source: J Clin Med. 2024 Jul 5;13(13):3952. doi: 10.3390/jcm13133952 (PMC11242427; doi:10.3390/jcm13133952)
Supplement: Supplementary file 1 [file jcm-13-03952-s001.zip › Supplementary Table S2.pdf]

Supplementary Table S2. Quality assessment of case series using JBI

Author, year	1	2	3	4	5	6	7	8	9	10	Overall
Choi, 2013	Y	Y	Y	Y	Y	Y	Y	Y	Y	Y	10
Ismat, 2019	Y	Y	Y	Y	Y	Y	N	Y	Y	Y	9
Lee, 2014	Y	Y	Y	Y	Y	N	N	Y	N	Y	7
Bernstein, 2018	Y	Y	Y	Y	Y	Y	Y	Y	Y	Y	10
Nagi, 2021	Y	Y	Y	N	N	N	Y	Y	Y	Y	7
Young, 2020	N	Y	Y	Y	Y	Y	Y	Y	Y	Y	9

Criteria used to rank the risk of bias

I) ≤49% = high risk of Bias

II) 50% and 69% = Moderate risk of Bias

III) Above 70% = low risk of Bias
